# Hedgehog signaling sensitizes Glioma stem cells to endogenous nano-irradiation

**DOI:** 10.18632/oncotarget.2123

**Published:** 2014-06-20

**Authors:** Agnieszka Morgenroth, Andreas T. J. Vogg, Katja Ermert, Boris Zlatopolskiy, Felix M. Mottaghy

**Affiliations:** ^1^ Department of Nuclear Medicine, University Hospital Aachen, RWTH Aachen University, Aachen, Germany; ^2^ Max Planck Institute for Neurological Research, Cologne, Germany; ^3^ Department of Nuclear Medicine, Maastricht University Medical Center, Maastricht, the Netherlands

**Keywords:** Hedgehog, glioma stem cells, Auger electron emitter, nano-irradiation, thymidine analogue

## Abstract

The existence of therapy resistant glioma stem cells is responsible for the high recurrence rate and incurability of glioblastomas. The Hedgehog pathway activity plays an essential role for self-renewal capacity and survival of glioma stem cells. We examined the potential of the Sonic hedgehog ligand for sensitizing of glioma stem cells to endogenous nano-irradiation. We demonstrate that the Sonic hedgehog ligand preferentially and efficiently activats glioma stem cells to enter the radiation sensitive G2/M phase. Concomitant inhibition of *de novo* thymidine synthesis with fluorodeoxyuridine and treatment with the Auger electron emitting thymidine analogue 5-[I-125]-Iodo-4′-thio-2′-deoxyuridine ([I-125]ITdU) leads to a fatal nano-irradiation in sensitized glioma stem cells. Targeting of proliferating glioma stem cells with DNA-incorporated [I-125]ITdU efficiently invokes the intrinsic apoptotic pathway despite active DNA repair mechanisms. Further, [I-125]ITdU completely inhibits survival of glioma stem cells *in vitro*. Analysis of non-stem glioblastoma cells and normal human astrocytes reveals that glioma stem cells differentially respond to Sonic hedgehog ligand. These data demonstrate a highly efficient and controllable single-cell kill therapeutic model for targeting glioma stem cells.

## INTRODUCTION

Human glioblastoma multiforme (GBM) evolving from the glial cells is the most common and at the same time most aggressive malignant primary brain tumor with an incidence rate of 2-3/100000 inhabitants [1]. After surgery usually adjuvant radiotherapy followed by chemotherapy with temozolomide is performed. Despite this aggressive approach the median survival is only about 15 month, if untreated the survival is between 3 to 4 months [2].

The high rate of recurrence of gliomas may be related to the existence of cancer stem cells (CSC) that are seemingly resistant to currently applied therapeutic regimens [3-5]. Surprisingly, the ionizing radiation therapy of glioblastoma was shown to enrich the proportion of CD133^+^ CSC population *in vitro* and *in vivo* [6]. Evidently, the remaining glioma stem cells (GSC) become highly radioresistant and tumorigenic by preferential activation of the DNA damage response. For sustained growth GSC require the Hedgehog (HH) signaling pathway [7]. This evolutionarily conserved signaling pathway serves critical functions in the regulation of organogenesis during embryogenesis as well as the maintenance of the tissue homeostasis and repair after injury in adult life [8]. The increased potential for tumor formation and self-renewal is partially regulated by the cross-talk between the HH pathway and the Phospoinositide 3-Kinase (PI3K)/Akt-Kinase pathway [9]. In this context, Wei et al. defined the functional significance of CD133 for HH signaling [10]. CD133 was shown to promote the tumorigenic capacity of GCS by activation of the PI3K/Akt pathway via the interaction with the regulatory subunit of PI3K p85. The elevated expression of the activating co-receptor Smoothened (Smo) and the transcription factor Glioma-Associated Oncogene homolog 1 (Gli 1) on the one hand, and the strongly reduced expression of the repressor receptors Patched 1 (Ptch1) and hedgehog-interacting protein (Hip) on the other hand, confer the GSC the unique self-renewal and tumorgenicity potential [7, 11, 12]. The deregulation of the HH pathway represses the retinoblastoma tumor suppressor-gene (Rb) and induces expression of the proto-onco gene *N-myc*, a key transcription factor which is overexpressed in malignant glioma cells [13]. Recent studies with cyclopamine, a Sonic hedgehog (SHH) inhibitor, have highlighted the importance of HH signals for growth and survival of CSC [14, 15]. Importantly, the HH signal has the potential to instruct the quiescent GSC to enter into the cell cycle. This in turn renders these cells sensitive to drugs targeting mitogenic signals and processes.

Due to the pronounced efficacy of cell killing, Auger emitter bearing nucleosides have been proposed as radiopharmaceuticals for systemic cancer endoradiotherapy [16]. Among them, the thymidine (Thd) analogue 5-Iodo-4'-thio-2'-deoxyuridine (ITdU) is characterized by efficient phosphorylation by the thymidine kinases and subsequent retention within the cell in a kinase-dependent manner. Due to the 4′-thio substitution ITdU displays resistance against enzymatic cleavage of the C–N glycosidic bond by thymidine phosphorylase (TP). Importantly, ITdU was shown to be rapidly incorporated into nuclear DNA through the thymidine salvage pathway providing a promising mechanism for delivering Auger radiation emitters such as I-123 and I-125 into tumor DNA [17, 18]. However, the application of ITdU is impaired by high background activity due to thymidylate synthase (TS) mediated deiodination of ITdU and generation of I-125 metabolites [17, 19]. The conditioning of tumor cells with a selective TS inhibitor fluorodeoxyuridine (FdUrd) efficiently blocked the dehalogenation of [I-125]ITdU and increased the substrate flux through the thymidine salvage pathway as indicated by the 18-fold increased DNA uptake of [I-125]ITdU, resulting in roughly 20-fold increased apoptosis induction in malignant cells [20]. Similarly, in an acute leukemia HL60 xenografted SCID mouse model, the FdUrd pre-treatment considerably increased the selectivity of ITdU towards tumor by increased cellular uptake and the incorporation into the DNA in malignant cells [18]. As a consequence, [I-125]ITdU preferentially induced apoptosis in tumor cells, whereas the viability of cells in normal proliferating tissues remained basically unaffected. Auger electron emitters incorporated into DNA are extremely toxic to the cell. This effect is due to the emission of high ionization density electron clouds that dominantly induce double strand breaks (DSB), which are highly cytotoxic forms of DNA damage. The accumulating damage significantly increases genetic instability and reduces the apoptotic threshold of the cell [21]. The reliance of stem cells on error-prone non-homologous end joining (NHEJ) would additionally boost the radiotoxic effect by allowing of miss incorporation of ITdU into the DNA during the damage repair process [22]. The properties of I-125 (high linear energy transfer (LET) and short path length) limit the cytotoxic effects to a sphere of a few nanometers in the immediate vicinity of the decay site. Cytosolic and extracellular decay of Auger emitters in contrast is 10-100 fold less radiotoxic than decay within DNA. These different effects account for the unique advantages of targeted nano-radiotherapy using Auger electron emitting drugs [23]. Recently, we presented a new promising approach involving radio-sensitization of myeloma stem-like cells by FdUrd pre-treatment followed by a DNA nano-irradiation by incorporated [I-125]ITdU [24]. FdUrd was shown to activate the relatively quiescent myeloma stem-like cells to enter cell cycling and to differentiate. Consequently, the acquired injury- and repair-activated state allowed complete eradication of myeloma stem-like cells.

Since the HH signaling acts as a mitogenic factor for glioma stem cells, we investigated the potential of SHH for sensitizing of highly therapy resistant GSC to an endogenous nucleoside analogue-based radiotherapy. This approach has the potential of an innovative therapeutic option for malignant brain tumors.

## RESULTS

### The HH pathway is active in a subpopulation of primary GBM

**Figure 1:**
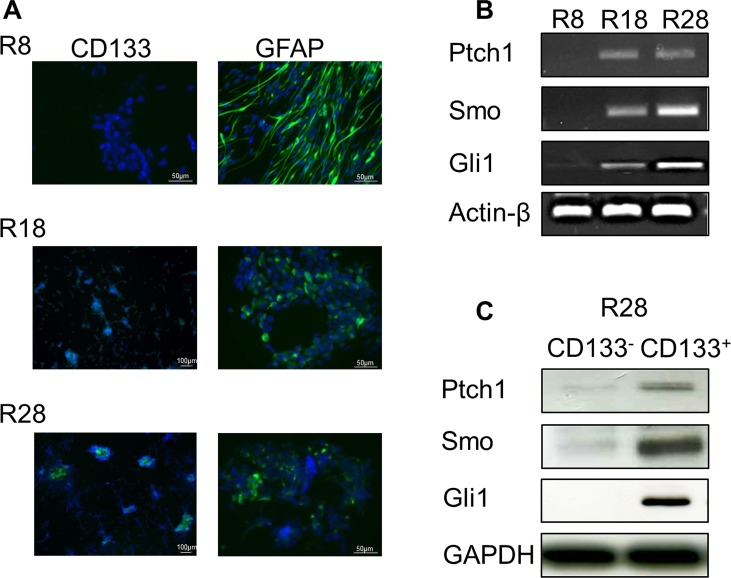
Characterization of CD133^+^ and CD133^−^ cells from human primary glioblastoma specimens (A) Representative pictures of CD133 (green staining) and GFAP (green staining) immunofluorescence staining in GBM cells R8, R18 and R28 (Hoechst nuclei staining in blue). (B) mRNA expression levels of Ptch1, Smo and Gli1 in R8, R18 and R28 GBM cells. Actin-β served as a loading control. (C) SDS-PAGE and Western blot analysis of HH pathway components Ptch1, Smo and Gli1 in separated CD133^−^ and CD133^+^ subpopulations of R28 cells. GAPDH served as a loading control.

To evaluate the HH pathway existence and activity in GSC, we examined the expression of pathway components in primary tumor samples R8, R18 and R28. The membrane-associated patched receptor 1 (Ptch1) and smoothened co-receptor (Smo) were detected exclusively in CD133^+^ cells containing tumor samples R18 and R28 using RT-PCR and SDS-PAGE Western blot analysis (Fig. 1). Correspondingly, both cells lines exhibited an expression of glioma-associated oncogene transcription factor (Gli1). Importantly, the expression of HH pathway components was restricted to the fraction of CD133^+^ GSC. Since Gli1 expression is consistently correlated with HH pathway activation, these data suggest that HH signaling is active in CD133^+^ cells containing tumor samples R18 and R28.

### SHH promotes proliferation of GBM-derived neurospheres

**Figure 2:**
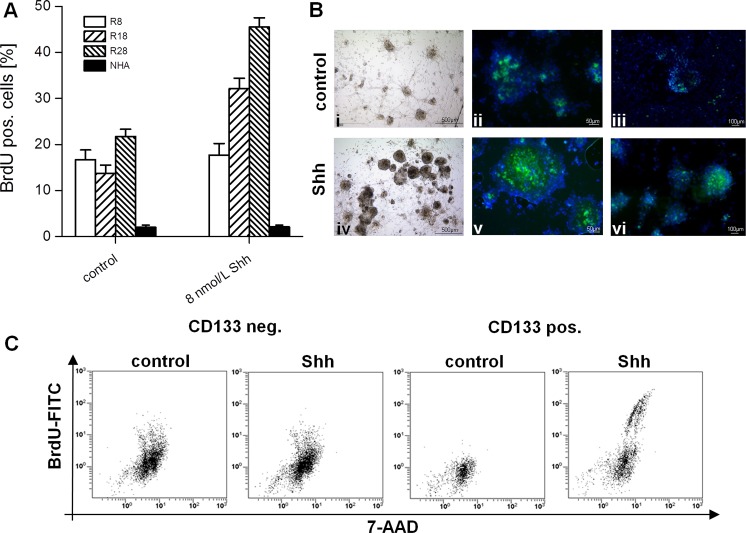
Effect of SHH stimulation on proliferation of glioma cells and normal astrocytes (A) Percentage of BrdU^+^ R8, R18, R28 cells and normal human astrocytes (NHA) after stimulation with SHH in comparison with unstimulated cells. Data are mean ± SD from three experiments. (B) Phase-contrast images (i, iv) and fluorescent images after staining with BODIPY-Cyclopamine (anti-Smo, green; ii, v) and anti-BrdU antibody (green; iii, vi) of unstimulated and SHH stimulated R28 neurospheres (Hoechst nuclei staining in blue). (C) Representative cytometries of anti-BrdU-FITC and 7-AAD (total DNA) double staining of unstimulated or SHH stimulated CD133^−^ and CD133^+^ R28 cells.

**Figure 3:**
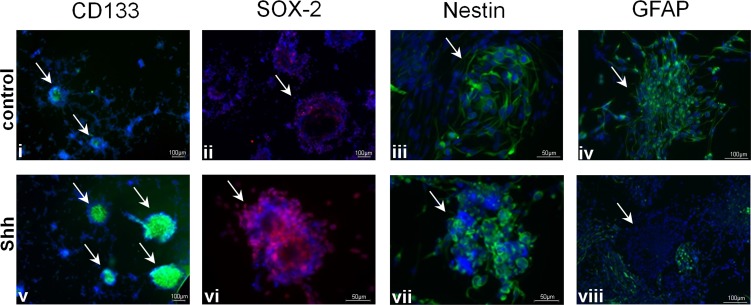
Effect of SHH stimulation on CD133^+^ GSC Representative fluorescent images after staining with anti-CD133 antibody (green; i, v), anti-SOX-2 antibody (red; ii, vi), anti-Nestin antibody (green; iii, vii) and anti-GFAP antibody (green; iv, viii) of unstimulated or SHH stimulated CD133^+^ R28 cells (Hoechst nuclei staining in blue). The neurospheres are marked by arrows.

To test for a possible role of HH-Gli signaling in GSC, we treated CD133^−^ und CD133^+^ cell cultures with SHH. Activation of HH pathway in R18 and R28 cells induced a marked proliferation. Addition of BrdU for 12h after 3d in culture showed an approximately twofold increase in the percentage of BrdU-positive cells (13.7%±1.8% vs. 32.1%±2.3% and 21.7%±1.6 vs. 45.5%±2.0% for R18 and R28, respectively) (Fig. 2A). By contrast, the CD133^−^ R8 cells did not respond to SHH stimulation (16.7%±2.1% vs. 17.7%±2.5%). Importantly, the degree of normal human astrocytic proliferation remained modest despite SHH stimulation. Remarkably, the HH-Gli signaling increased both, number and volume of neurospheres, which became strongly positive for SHH receptor Smo (Fig. 2B). Moreover, the induced mitotic activity was restricted to the neurospheres, which reflect the aggressiveness of CSC and are considered as a significant prognostic factor for tumor progression [25]. Consistent with the RT-PCT and Western Blot analysis of HH signal cascade components, the stimulating effect of SHH was detectable solely in the CD133^+^ glioma population, the proliferation index in CD133^−^ cells remained unaffected (Fig. 2C). Additionally, SHH activated CD133^+^ glioma cells demonstrated an increased expression pattern of stemness markers like CD133, SOX-2 and Nestin (Fig. 3). Simultaneously, the number of GFAP-expressing cells in the neurospheres declined markedly.

### HH activation increases the cellular uptake and the DNA-incorporation rate of [I-125]ITdU in GSC

**Figure 4:**
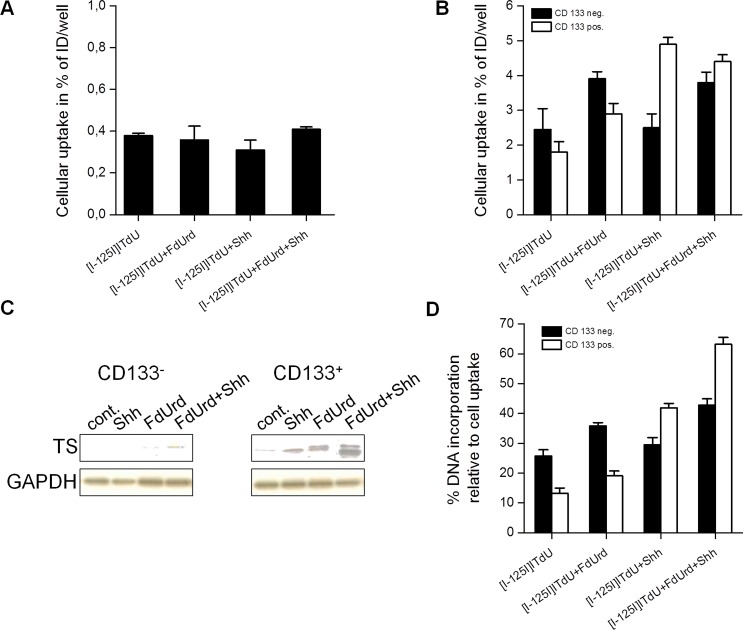
Effects of FdUrd and SHH conditioning on cellular uptake and DNA-incorporation of [I-125]ITdU in normal astrocytes and CD133^−^ and CD133^+^ R28 glioma cells (A) Cellular uptake of [I-125]ITdU (% of incubated dose (ID)/well) in normal human astrocytes in dependency on FdUrd and SHH stimulation. Data are mean ± SD from three experiments. (B) Cellular uptake of [I-125]ITdU (% of incubated dose (ID)/well) in CD133^−^ and CD133^+^ R28 cells in dependency on FdUrd and SHH stimulation. Data are mean ± SD from three experiments. (C) SDS-PAGE and Western blot analysis of TS expression in CD133^−^ and CD133^+^ R28 cells with and without conditioning with FdUrd and SHH. GAPDH served as a loading control. (D) DNA incorporation of [I-125]ITdU (% DNA association relative to total cellular uptake) in CD133^−^ and CD133^+^ R28 cells in dependence on FdUrd and SHH stimulation. Data are mean ± SD from three experiments.

Normal human astrocytes exhibited virtually no cellular uptake of [I-125]ITdU under all tested conditions (Fig. 4A). In glioma cells, without stimulation only a small cell portion displayed uptake of [I-125]ITdU. Generally, in accordance with the TS expression pattern in CD133^−^ and CD133^+^ glioma cells, the conditioning with FdUrd and SHH synergistically enhanced the incorporation of [I-125]ITdU in GSC, while in CD133- glioma cells only an additive effect was observed (Fig. 4B,C). Short term pre-exposure to FdUrd increased the cellular uptake and retention of [I-125]ITdU in TS-negative CD133^−^ glioma cells (2.4%±0.6% vs. 3.9%±0.4%). This effect was less pronounced in CD133^+^ glioma cells (1.8%±0.3% vs. 2.9%±0.2%). Consistent with our stimulation study, the capacity of SHH to modulate proliferation of GSC resulted in an approx. twofold increase of [I-125]ITdU uptake in CD133^+^ cells (4.9%±0.6% vs. 2.5%±0.6% in CD133^+^ and CD133^−^ glioma cells, respectively). The combination of FdUrd and SHH only moderately influenced the accumulation of [I-125]ITdU. Whereas the HH pathway activation did not affect the cellular uptake of [I-125]ITdU remarkably, the treatment with SHH dramatically changed the incorporation rate of tracer into DNA (Fig. 4D). The proportion of GSC that inserted [I-125]ITdU into the DNA increased from 13.2%±1.8% to 41.9%±1.5%. Conversely, FdUrd pre-treatment elevated the DNA incorporation of [I-125]ITdU exclusively in CD133^−^ glioma cells. Importantly, simultaneous activation of HH pathway and inhibition of thymidine *de novo* synthesis pathway showed a synergistic effect on [I-125]ITdU incorporation in glioma cells (63.2%±2.3% and 42.8%±2.1% in CD133^+^ and CD133^−^ cells, respectively).

### SHH promotes [I-125]ITdU mediated apoptosis of GSC through a caspase-dependent mechanism

**Figure 5:**
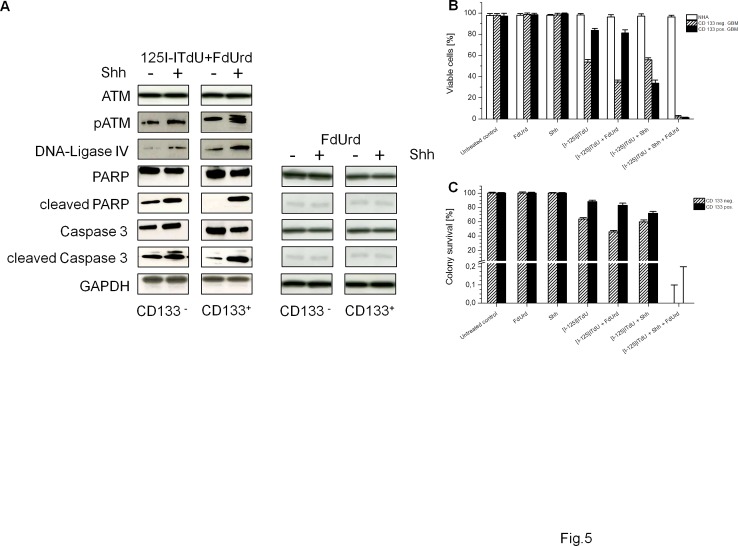
Effects of [I-125]ITdU on survival of CD133^−^ and CD133^+^ R28 cells (A) SDS-PAGE and Western blot analysis of the activation of checkpoint response, DNA repair mechanisms and induction of apoptosis in response to [I-125]ITdU with FdUrd w/o SHH in CD133^−^ and CD133^+^ R28 cells (left). SDS-PAGE and Western blot analysis of apoptosis marker in CD133^−^ and CD133^+^ R28 cells after treatment with FdUrd w/o SHH (right). GAPDH served as a loading control. (B) The percentage of CD133^−^ and CD133^+^ R28 viable cells after incubation with [I-125]ITdU in dependency on FdUrd and SHH stimulation. Data are mean ± SD from three experiments. (C) Clonogenic survival of CD133^−^ and CD133^+^ R28 cells after incubation with [I-125]ITdU in dependency on FdUrd and SHH stimulation. Data are mean ± SD from three experiments.

Since the DNA damage checkpoints are essential for cellular radiosensitivity [26], we determined the activation of the ataxia-telangiectasia-mutated protein (ATM) after incubation with [I-125]ITdU in CD133^+^ and CD133^−^ glioma cells. In both cell subpopulations, DNA damage induced by [I-125]ITdU mediated nano-irradiation potentially initiated activating phosphorylation of ATM (Fig. 5A). Stimulation with SHH remarkably potentiated the checkpoint activation in CD133^+^ GSC. Moreover, expression of DNA-Ligase IV, a protein involved in the repair of double strand DNA breaks, was clearly increased in the CD133^+^ GSC. Neither CD133^+^ cells nor CD133^−^ cells underwent apoptosis after stimulation with FdUrd and SHH alone. The intrinsic apoptotic pathway activation in CD133^+^ GSC by [I-125]ITdU was found to depend on SHH stimulation. The exposure to [I-125]ITdU alone was not sufficient to trigger the cell death, as indicated by decreased activation of PARP and Caspase 3. Consequently, in the absence of SHH, more than 80% of CD133^+^ cells remained viable after exposure to [I-125]ITdU (Fig. 5B). By contrast, about 50% of CD133^−^ cells were determined as apoptotic. Consistent with DNA-incorporation rate of [I-125]ITdU, the pre-treatment with FdUrd alone was sufficient to increase the depletion of the CD133^−^ cells but not of CD133^+^ cells (46.3%±1.8% vs. 65.2%±1.6% and 16.3%±2.3% vs. 19.2%±2.0% for CD133^−^ and CD133^+^ cells, respectively). The activation of HH pathway diminished more than fourfold the percentage of viable CD133^+^ cells, whereas no additive effect was observed in CD133^−^ cells. Importantly, simultaneous treatment with SHH and FdUrd completely eliminated both cell fractions. The viability of NHA remained unaffected.

### [I-125]ITdU mediated elimination of SHH sensitized GSC abolishes the clonogenic recovery of tumor cells

Neither single treatment with FdUrd nor with SHH was sufficient to sensitize the CD133^+^ GSC to [I-125]ITdU induced cell death (Fig. 5C). The activation of HH pathway prior to short-term incubation with FdUrd was indispensable to achieve a complete inhibition of clonogenic CD133^+^ GSC growth by [I-125]ITdU emitted nano-irradiation. Pre-treatment with FdUrd or SHH alone was completely non-toxic.

## DISCUSSION

In the current study we evaluated a two-step killing strategy of glioblastoma multiforme stem cells showing extraordinary efficiency. It is for the first time that a direct and selective activation of a small subpopulation of highly therapy resistant CSC [27] could be implemented as a first step towards selective nano-irradiation induced apoptosis. In case of recurrence currently employed monotherapies usually fail to achieve a complete cancer remission, therefore multimodal approaches may represent more effective treatments. In this regard, the molecular targeting of development- and maintenance-associated pathways in CSC can represent an innovative strategy for improving the outcome. Since the genetic abnormalities within the HH pathway are thought to be one of the key mechanisms contributing to the initiation of GSC [28], we decided to stimulate them via a SHH ligand.

**Figure 6:**
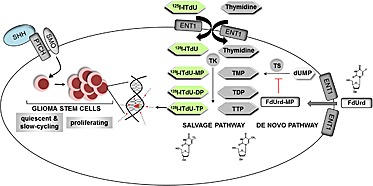
A two-step killing strategy of glioblastoma multiforme stem cells Glioma stem cells are activated by HH signaling pathway to enter mitosis. The proliferating glioma stem cells incorporate the radiolabelled thymidine analogue [I-125]ITdU into the DNA via the salvage pathway. This effect is further enhanced by simultaneous FdUrd mediated inhibition of the *de novo* pathway of thymidine synthesis.

Our study shows that activating HH signaling using SHH initiates the GSC to enter mitosis and by this they become highly sensitive for nano-irradiation (Fig. 6). We could demonstrate that the components required to transduce the HH signal are present in CD133^+^ GSC but not in CD133^−^ glioma cells. This was accomplished by strong expression of Gli1, a transcription factor which is considered to be an essential hallmark of aberrant HH pathway activation [29]. Moreover, the CD133^+^ cells showed characteristics consistent with CSC, namely neurospheres formation, expression of neural/cancer stem cell markers (CD133, Nestin, SOX-2) and differentiation marker (GFAP) [3]. Consistent with mRNA and protein expression analysis, only GSC responded to SHH stimulation by increased proliferation. Naturally, the SHH ligand acts autocrine and paracrine as a potent morphogen and mitogen for tissue-residual stem/progenitor cell in a time- and concentration-dependent manner [30]. In the adult brain, most neural stem cells (NSC) are located in the subventricular zone [31]. Importantly, despite the target overlay of SHH mitogen action, in the NSC the redundant activity of HH pathway proteins is governed in part by their expression patterns and diverse regulator mechanisms. First, in regions with ongoing adult neurogenesis, cell proliferation is balanced by expression of a negative regulatory feedback loop. More specifically, under normal physiological conditions the HH pathway remains dormant through the enhanced expression of an endogenous HH inhibitor, hedgehog-interacting protein (Hip) [12]. This molecular event impedes the binding of SHH to the Ptch1 receptor on the NSC and inhibits the signal transduction. Conversely, the GCS was shown to exhibit an increased expression of Smo, the activating receptor of the HH pathway. Downstream the HH pathway, the NSC were shown to express the repressor transcriptional factor Gli3, whereas GSC overexpress the activation factor Gli1 [30]. Furthermore, the SHH expression is differentially regulated by epigenetic mechanisms. In GSC, an expression of O6-methylguanine-DNA- methyltransferase (MGMT) is virtually absent [32]. Thereby, the hypomethylation status of the SHH region promoter would yield an up-regulation of the SHH expression in CD133^+^ cells. Consistent with these observations, stimulation with SHH would preferentially address GSC, and the application of SHH might represent a potential strategy to activate quiescent GSC prior to nano-irradiation. In our study, the expanded neurospheres exhibited high expression patterns for Smo and CSC markers, and mitogenic activity. Moreover, in the presence of exogenous SHH, the GSC displayed only a weak expression of GFAP. These data suggest that stimulation via HH pathway promotes expression of GSC markers and prevent cell differentiation.

Since proliferating cells are excellent therapy targets, we decided to investigate the efficiency of the Auger-electron emitting thymidine analogue [I-125]ITdU for eradication of GSC. Auger electron therapy is a promising form of targeted endoradiotherapy, which allows irradiation of single cells due to the unique physical characteristics of emitted Auger electrons, namely the short range and high local toxicity. Several pre-clinical and clinical studies emphasize the benefit of the use of Auger electron emitting radiopharmaceuticals [16]. Correspondingly to the proliferation analysis, stimulation with SHH increased the cellular uptake and DNA-incorporation of [I-125]ITdU in GSC. This effect was less pronounced in SHH-unresponsive CD133^−^ glioma cells. The inhibition of the *de novo* thymidine synthesis pathway by FdUrd alone addressed more effectively the CD133^−^ glioma cells. This is likely due to the effect of FdUrd on the redistribution of nucleoside transporters hENT1, which in turn results in enhanced nucleosides influx [33]. This observation together with the higher mitotic activity, which is characterized by a massive (10-30-fold) expansion of dNTPs pool sizes [34], might explain an affected cellular uptake and DNA-incorporation of [I-125]ITdU in CD133^−^ glioma cells. The exclusive expression of TS, in GSC supports the function of TS during the oncogenesis, since TS overexpression was shown to contribute to neoplastic transformation in NIH/3T3 fibroblasts [35]. Moreover, TS catalyzes an important step of the *de novo* synthesis independently from the extracellular thymidine supply. Thus, the existence of TS might confer GCS an autonomous character as defined for highly resistant cancer stem cell. Interestingly, stimulation of the HH pathway increased the expression of TS. Consistent with defined relief of TS translational repression by TS inhibitor [36], the enzyme production was further increased by treatment with FdUrd. The main consequence of TS inhibition relies on significant depletion of intracellular thymidine triphosphate (TTP) which strongly affects the production and balance of DNA precursor pools. Therefore, the activation of HH pathway combined with inhibition of the *de novo* thymidine synthesis resulted in remarkably increased incorporation of [I-125]ITdU in to the DNA of GSC (from 13.2%±1.8% to 63.2%±2.3%).

The DNA-incorporated [I-125]ITdU was shown to induce extensive DNA damages, mostly by generation of DSB (up to 32 breaks per decay) [37]. DNA damage checkpoint responses play an essential role in cellular radiosensitivity, and their enhanced activation in response to radiation induced DNA damage was observed within the CSCs subpopulation [6]. Similarly, in this study the CD133^+^ glioma cells were more resistant to [I-125]ITdU emitted nano-irradiation than the non-stem glioma cells. [I-125]ITdU activated more potently the DNA damage checkpoint response as detected by phosphorylation of ATM in GSC. After HH pathway activation, GCS showed strongly enhanced checkpoint activation, due to the increased DNA-incorporation of [I-125]ITdU. As a result, GSC were more efficient in initiation of the NHEJ DNA repair mechanism, the predominant mechanism in DSB repair in CSC [22]. Consequently, as indicated by decreased activation of PARP and Caspase 3, after exposure to [I-125]ITdU GSC showed lower rate of apoptosis and increased survival than the non-GCS. Evidently, owing to the combined conditioning with SHH and FdUrd the GSC became sensitive for [I-125]ITdU emitted nano-irradiation. Despite the activation of DNA damage repair mechanisms, the majority of cells was primed for apoptosis resulting in a complete inhibition of clonogenic growth. Our *in vitro* data clearly show the selective and efficient targeting of activated GSC by nano-irradiation. Regarding an *in vivo* application of FdUrd and [I-125]ITdU, we previously demonstrated a massive apoptosis induction in the tumor tissue, and only moderate damage in normal proliferating tissues like the spleen and small intestine crypt cells [18]. Considering the unique characteristic of the Auger electron emitters (e.g. single-cell kill), the low mitotic activity of normal brain cells [38] and the different expression profile of HH pathway activating and repressing factors in GSC and NSC [12], we suggest that the novel treatment strategy might eliminate GSC without causing severe radio toxicity in normal tissues. In a clinical context, the crossing of the blood-brain barrier (BBB) challenges the efficiency of this therapeutic approach since thymidine and its analogues have a low BBB permeability [39]. Considering the BBB-related molecular alterations in the capillary endothelial cells vascularizing glioblastoma [40], we speculate that [I-125]ITdU will be able to reach the main bulk of gliomas through the disrupted BBB. The difficulty of treatment is how to reach GSC in the infiltration zone where the BBB is not or less altered. For this approach, a nano-scaled drug delivery system (e.g. functionalized nanoparticles [41]) allowing for [I-125]ITdU to be efficiently transported across the BBB for targeting of GSC could be a solution.

Our results argue for the potential of SHH as a sensitizer for [I-125]ITdU emitted nano-irradiation. Given that the activation via the HH pathway induced proliferation of GSC and showed additive and synergistic effects with FdUrd, a combined strategy with SHH and FdUrd might provide an innovative and potential strategy for complete elimination of GCS.

## MATERIALS AND METHODS

### Chemicals

Chemicals and solvents were purchased from Sigma-Aldrich (St. Louis, Missouri, USA) and Merck (Germany) or otherwise as indicated. All reagents and solvents were of the highest commercially available purity grade. No-carrier-added (n.c.a.) sodium [I-125]iodide was obtained from PerkinElmer (Waltham, Massachusetts, USA). The precursor of [I-125]ITdU, precursor 5-(trimethylstannyl)-4'-thio-2'-deoxyuridine, CAS no. 444586-71-4, and the unlabeled reference standard, 5-iodo-4'-thio-2'-deoxyuridine (ITdU) CAS no. 134699-95-9, were synthesized as previously reported [17]. Used water and acetonitrile for reagents were from Merck.

### Radiochemistry

Production of n.c.a. [I-125]ITdU was performed as follows. 16 μL Chloramine T (Sodium N-Chloro-p-toluenesulfonamide trihydrate, CAS no. 7080-50-4, 2.4 mM in H_2_O:CH_3_CN = 2:1) were added to a mixture of 17 μL phosphate buffer (0.2 M, pH 2.0, in H_2_O: methanol = 7:3), 3 μL precursor solution (123 mM in H_2_O: methanol = 1:2), and 10 μL n.c.a. [I-125]NaI solution in 0.05 M NaOH. Labelling reaction completed within 10 minutes at room temperature and was stopped by addition of 10 μL 100 mM sodium thiosulfate. This mixture was transferred to the HPLC port and reactor and injection syringe were finally flushed with 30 μL 10 % EtOH aq; thus an entire volume of 86 μL was injected. The product was purified by HPLC with UV at 265 nm and gamma detection (NaI detector, 13-1600 keV) using a MultoKrom 100-5 C4 (250 ×4 mm) reversed phase column (CS-Chromatography, Germany) eluted with 15% ethanol aq at a flow rate of 1 mL/min. Retention times were: 3.8 minutes for [I-125]iodide, 7.0 minutes for [I-125]ITdU. Product volume was 1.0 ± 0.2 mL. Quality control was performed by analytic HPLC with UV and gamma detection (column: LiChrospher 100 RP-18 5μ-EC, 125×4 mm, CS-Chromatography), eluent: 0.05 M phosphate buffer (pH 3.6) containing 12% methanol by volume, and flow rate: 1 mL/min, retention times: 1.9 minutes for [I-125]iodide, 9.0 minutes for [I-125]ITdU). Total radiochemical yields were 83 ± 8%. Radiochemical purities were > 98%. Molar concentrations of prepared n.c.a. [I-125]ITdU solutions in 15% ethanol aq. were 3 μM (+/- 10%) with typical activity concentrations of 50 MBq/mL (with batch variations +/- 10 MBq/mL) and corresponding specific activities of 20 MBq/μmol.

### Culture of primary GBM cells and spheres

Human primary GBM cells R8 (CD133^−^), R18 and R28 (CD133^+^) were kindly provided by Dr. Dagmar Beier (Department of Neurology, University Aachen, RWTH, Germany). The procedure of generation of primary GBM cells and histological classification were described by Beier et al [32, 42]. GBM cells were cultured in stem cell-permissive DMEM/F12 medium (PAN, Germany) supplemented with human recombinant epidermal growth factor, human recombinant basic fibroblast growth factor (both PeproTech, Rocky Hill, New Jersey, USA) and human leukemia inhibitory factor (Active Bioscence, Germany), 20 ng/mL of each, and 2% B27 (Gibco, Life Technologies, Carlsbad, California, USA) in non-tissue treated plates. These culture conditions were formulated to maintain the genetic and morphologic characteristics of primary tumor [43, 44]. For treatment, the cells were incubated in stem cell-permissive medium supplemented with SHH (8nM; R&D Systems, Minneapolis, Minnesota, USA). For isolation of CD133 positive stem cells, the magnetic CD133/1 Micro Beads (Miltenyi Biotech, Germany) were used.

### Culture of normal human astrocytes

Primary normal human astrocytes were obtained from Lonza. Cells were cultured in astrocytes medium supplemented with 0.1% rhEGF, 0.25% Insulin, 0.1% Ascorbic Acid, 0.1% GA-1000, 1% L-Glutamine, and 3% FBS (all supplements from Lonza, Germany).

### Flow cytometry and immunocytochemistry

The phenotype was investigated by staining with primary anti-CD133/2 (Miltenyi Biotech, 1:10), anti-glial fibrillary acidic protein (GFAP, BD Pharmingen, San Diego, California, USA, 1:10), anti-Nestin (Biozol, Germany, 1:200), anti-SOX-2 (Abcam, UK, 1:1000), and corresponding fluorescence labeled secondary antibodies (Abcam, Cell Signaling Technology). The isotype antibodies were used as a negative control. The proliferation status was determined using a BrdU and 7-ADD (7-amino-actinomycin D, total DNA) staining (BD Pharmingen) according to the manual instruction. To determine apoptosis, the cells were lysed with Nicoletti buffer containing 0.1% sodium citrate, 0.1% Triton X-100 and propidium iodide (50 mg/mL). The median fluorescence index (MFI) was evaluated by flow cytometry (FACS, Cytomics FC 500, Beckman Coulter, Germany). The FACS data were analyzed using CXP Software (Beckman Coulter). For fluorescence microscopy analysis the GBM cells were plated onto poly-lysine coated coverslips and fixed with 4% PFA. The SMO expression was investigated by incubation with BODIPY-Cyclopamine (5 nmol/L, Toronto Research Chemicals Inc., Canada) for 1 hour. Nuclei were counterstained using Hoechst33342 (1 mg/mL, Sigma-Aldrich), before being examined by fluorescence microscopy (Axio Scope A1, ZEISS, Germany).

### Cellular uptake and DNA incorporation of [I-125]ITdU

For uptake experiments, the stimulation with SHH (8 nM) was initiated 48 h prior the nano-irradiation. As the second step, the cells were pre-treated for 1 h with FdUrd (1 μM), washed with PBS and cultured for further 16 h in medium supplemented with SHH. Exponentially growing cells (1 x 10^4^/well) were incubated for 96 h with 200 kBq of [I-125]ITdU. Thereafter, they were washed three times with PBS and the intracellular accumulated radioactivity was assessed by a gamma counter (Wizard^2^, PerkinElmer). After measurement, DNA was extracted using the DNeasy Tissue Kit (Qiagen, Germany). Incorporated radioactivity was measured in a gamma counter. The experiments were carried out three times in triplicates.

### SDS-PAGE/Western blot analysis

For SDS-PAGE/Western blot analysis, the protein lysates were prepared by lysis with Tris-HCl buffer, NP-40 (1 %), PMSF (1 mM) and inhibitor cocktail (Roche, Switzerland). Protein samples were separated by 4-20% SDS-PAGE (BioRad, Berkeley, California, USA) and transferred onto PVDF membrane. Protein detection was performed with polyclonal antibodies: anti-thymidylate synthase (Abcam, 1:200), anti-ATM (Abcam, 1:2000), anti-phospho (S1981) ATM (Abcam, 1:1000), anti-DNA Ligase IV (Abcam, 1:500), anti-PARP (Cell Signaling, 1:1000), anti-cleaved PARP (Cell Signaling, 1:1000), anti-Caspase-3 (Cell Signaling, 1:1000), anti-cleaved Caspase-3 (Cell Signaling, 1:1000). The secondary anti-rabbit IgG coupled to HRP (Cell Signaling, 1:1000) was visualized with enhanced chemiluminescence (ECL+, GE Healthcare, UK). Equal protein loading was controlled using GAPDH specific antibody (Ambion, Life Technologies, Carlsbad, California, USA) and secondary goat anti-mouse IgG linked to HRP (Abcam). The experiments were carried out three times in triplicates.

### PCR validation of gene expression

Total RNA was extracted from untreated and treated cells using an RNAeasy kit (Qiagen). Reverse transcription was carried out using Advantage RT-for-PCR kit (Clontech, Mountain View, California, USA). RNA and cDNA were quantified by using a BioPhotometer (Eppendorf, Germany). For PCR analysis gene-specific primers were used at 10 pmol per reaction: Gli-1 forward primer 5'−CGGGGTCTCAAACTGCCCAGCTT−3'; Gli-1 reverse primer 5'−GGCTGGGTCACTGGCCCTC−3'; Ptch1 forward primer 5'−TTTGGACTGCTTCTGGGAAGGG−3'; Ptch1 reverse primer 5'−TTTTTGTTGGGGGCTGTGGC−3'; Smo forward primer 5'−CAGGACATGCACA GCTACATCG−3', Smo reverse primer 5'−CCACAAAGAAGCACGCATTGAC−3'; Actin-β forward primer 5′-CCCAGCACAATGAAGATCAA-3′, Actin-β reverse primer 5′-GATCCACACGGAGTACTTG-3′. PCR was performed using Advantage® cDNA PCR kit (Clontech) for 30 cycles. PCR products were resolved on a 1.2% agarose gel, stained with ethidium bromid, and bands were detected using the ImageQuant LAS 4010 camera system (GE Healthcare). The experiments were carried out three times in triplicates.

### Clonogenic assay

To measure the clonogenic survival, the treated cells (accordingly the treatment schedule described above) were harvested, counted and platted in growth medium with 1*10^4^ cells/ well. The plates were incubated for 3 weeks at 37°C and 5% CO_2_. Following incubation, the medium was removed and colonies were fixed and stained with 0.5% methylene blue (dissolved in 50% ethanol). Colonies containing ≥ 50 cells [45] were counted on an invert microscope (IT400 Trino Plan, VWR International, Radnor, Pennsylvania, USA). To determine the surviving fractions, the number of colonies after each treatment was normalized to that of the untreated control group. The experiment was carried out three times in triplicates.

### Statistical analysis

Cellular uptake with DNA-incorporation experiments, all FACS analyses, and clonogenic assays were performed in triplicate and by repeating independent blocks of experiments including all appropriate controls. Data are presented as mean ± standard deviation. The percentage of specific cell death was calculated as 100 × (experimental dead cells [%] – spontaneous dead cells in medium [%])/(100% – spontaneous dead cells in medium [%]).
